# A Physiologically Based Pharmacokinetic Model for Studying the Biowaiver Risk of Biopharmaceutics Classification System Class I Drugs With Rapid Elimination: Dexketoprofen Trometamol Case Study

**DOI:** 10.3389/fphar.2022.808456

**Published:** 2022-02-10

**Authors:** Xian Zhang, Xuxiao Ye, Kuan Hu, Wenping Li, Wenqian Li, Qingqing Xiao, Lin Chen, Jin Yang

**Affiliations:** ^1^ Center of Drug Metabolism and Pharmacokinetics, China Pharmaceutical University, Nanjing, China; ^2^ Mosim Pharmaceutical Technology Co., Ltd., Shanghai, China

**Keywords:** gastric emptying, bioequivalence, physiologically based pharmacokinetic model, dexketoprofen trometamol, biowaiver

## Abstract

Biowaiver based on the biopharmaceutics classification system (BCS) has been widely used in the global market for the approval of new generic drug products to avoid unnecessary *in vivo* bioequivalence (BE) studies. However, it is reported that three out of four formulations of dexketoprofen trometamol (DEX) tablets (BCS class I drug) failed the first BE study. The aim of this study was to determine whether the current biowaiver standard is reasonable for DEX. Thus, we successfully established a physiologically based pharmacokinetic (PBPK) model for DEX and examined the effects of dissolution, permeability, and gastric emptying time on DEX absorption under BCS-based biowaiver conditions using sensitivity analyses. Parameter sensitivity analysis showed that the dissolution rate in pH 1.2 media, permeability, and liquid gastric emptying time were sensitive parameters of C_max_. Therefore, gastric emptying variation was introduced into the PBPK model, and virtual BE studys were conducted on original research formulation and the formulation of the boundary dissolution rate (f2 = 50) prescribed by the biowaiver guideline. The virtual BE results showed dissolution rate changes within the biowaiver range will not cause high non-BE ratio, indicate waive of DEX generic drugs would not lead the risk of C_max_ when generic products satisfy the requirements of biowaiver guideline. However, the effect of excipients on gastric emptying as a sensitive factor needs to be further studied when the rapid elimination of BCS class I drug is biowaived.

## Introduction

Bioequivalence (BE) studies are widely used to evaluate therapeutic equivalence between generic and original drugs. Nevertheless, Clinical BE studies of biopharmaceutics classification system (BCS) class I and class III drugs are unnecessary because of their low risk of bioInequivalence (BIE). Thus, a waiver of *in vivo* BE studies based on the BCS for immediate-release solid oral dosage forms has been proposed and widely accepted by regulators (EMA, 2010; FDA, 2017; ICH, 2019). The benefit of the biowaiver is not only to reduce research cost by replacing human research with *in vitro* experiments but also, more importantly, promote the development of new compounds that would benefit public health.

The biowaiver of oral drugs based on the BCS classification is an effective scientific regulatory tool for granting biowaivers for solid oral immediate-release drug products following *in vitro* tests (Fagerholm, 2007). Based on the scientific principles of BCS, *in vivo* differences in the rate and extent of drug absorption between two pharmaceutically equivalent solid oral products may be caused by differences in the *in vivo* dissolution of the drug. As most drugs cannot be absorbed in the stomach, when an immediate-release oral dosage form dissolves faster than the gastric emptying rate, the rate and extent of drug absorption may not be related to drug dissolution. Therefore, for BCS class I drugs, when the general product meets the requirement of more than 85% release within 30 min and a similar dissolution profile to that of the reference product, unless the general product contains excipients that affect the absorption of active ingredients, the BE test in humans can be waived.

However, it has been reported that three out of four formulations of the BCS class I drug dexketoprofen trometamol (DEX) tablets failed the first BE study in Spain (Garcia-Arieta et al., 2015). Four of the eight BE experiments conducted on generic drug products showed non-BE results. Among them, the geometric mean ratio (GMR) of C_max_ of the two failed BE experiments exceeds the equivalent interval (80–125%), GMR of the other two failed experiments are within the equivalent interval but the 90% confidence interval (CI) exceeds 80–125%. Therefore, in order to analyze the reasons for the non-BE of the rapidly eliminated BCS class I drugs DEX and to control the risk of biowaiver, we tried to establish a human physiologically based pharmacokinetic (PBPK) model of DEX.

Dexketoprofen is a nonsteroidal anti-inflammatory drug that exerts analgesic and anti-inflammatory effects by inhibiting prostaglandin synthesis (Barbanoj et al., 2001). DEX is a highly aqueous soluble salt of dexketoprofen. It has the highest solubility in buffer media with a pH 6.8 and the lowest solubility in 0.1 N HCl (0.226 mg/ml), but it is still high enough to meet the solubility standard for BCS class I drugs (Sweed et al., 2019). The apparent permeability coefficient (*P*_app_) of DEX is 81.6 × 10^–6^ cm/s in Caco-2 cells (Laitinen et al., 2003), and the effective intestinal permeability (*P*_eff_) of DEX in humans is 3.37 cm/h, which indicates high permeability. Kortejärvi and co-workers (Kortejärvi and Aurtti, 2007) showed that BCS class I drugs with rapid elimination have a higher risk to fail in a BE study than BCS class III drugs. When comparing the solid dosage form with an absorption rate of 8 h^−1^ and an elimination rate of 0.9 h^−1^ with the oral solution of BCS class I drugs, the difference in peak plasma concentration (C_max_) even reached 25%. The time to maximal plasma concentration (T_max_) value of solid dosage form DEX after oral administration at a dose of 37 mg (corresponding to 25 mg of dexketoprofen) was approximately 0.5 h (Barbanoj et al., 2001). A human study showed that its elimination was rapid, with a half-life of approximately 1.3 h, which indicated that DEX is a typical BCS class I drugs with rapid elimination (solid dosage form) (Kortejärvi and Aurtti, 2007). To reduce the clinical failure in the drug development process and ensure the safety and effectiveness of BCS-based biowaiver of drugs, it is necessary to identify whether the current biowaiver criteria are overly conservative or venturesome for DEX. If DEX is not approved for a biowaiver, this may potentially affect policy change on biowaivers for BCS class I drugs.

**FIGURE 1:**
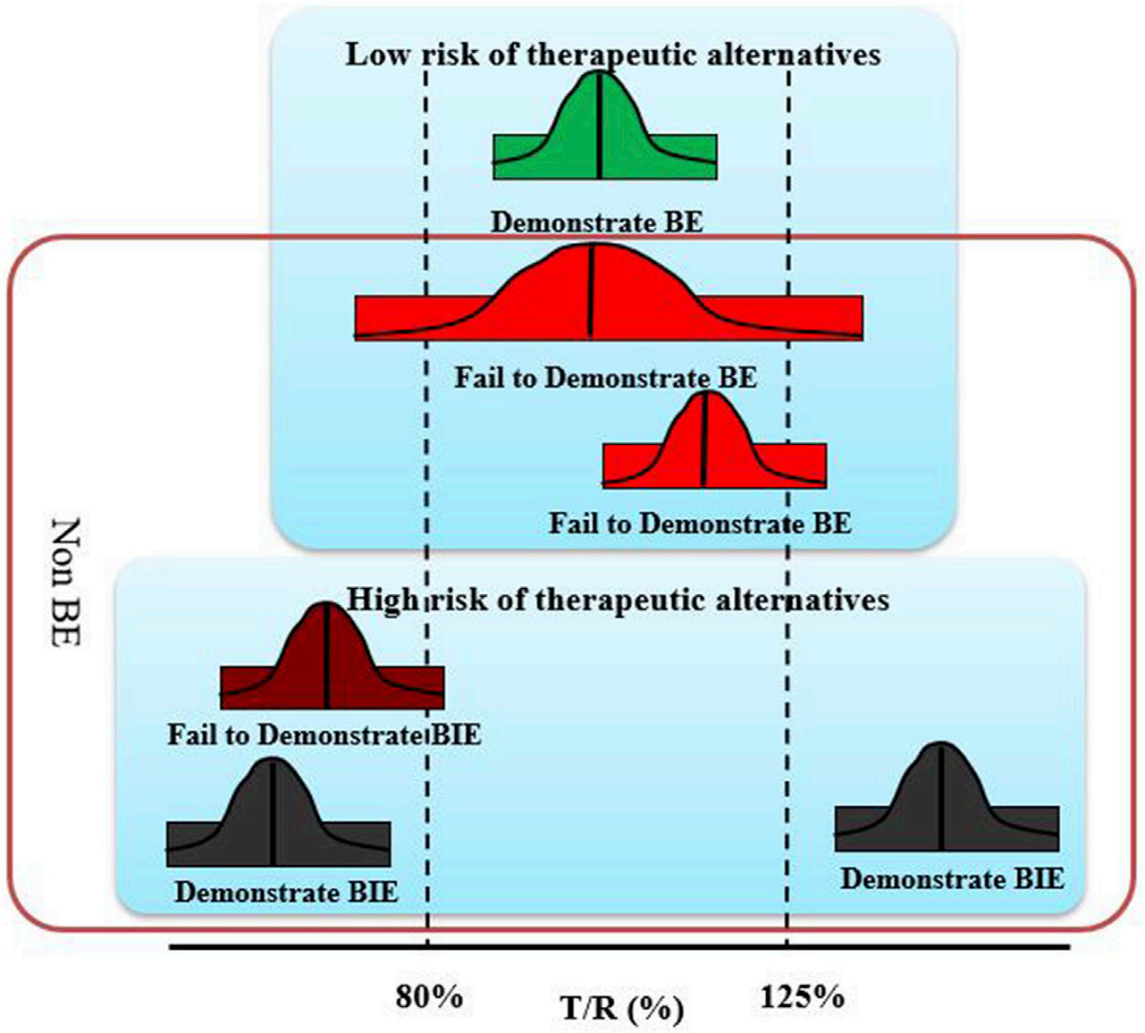
Schematic diagram of four equivalent test results.

In order to in-depth analysis of the reasons for the non-BE of DEX, we refined the BE results. A failure of BE experiments could be explained by several reasons, including significant formulation differences, improper clinical experiment control, unproper bio-analysis methods, and unreasonable experimental design, such as insufficient subjects. Among them, the inherent differences in the formulation truely affects the therapeutic alternatives of the drug. The GMR is a formulation issue, thus, according to whether the GMR fell within 80–125%, the results were simply divided into “low risk of therapeutic alternatives” and “high risk of therapeutic alternatives”. This is because the GMR of fail to demonstrate BE studies close to 1 is related to small differences in the formulation; however, as it is difficult to prove, a result that failed to show BE was obtained. The reference and test products were considered bioequivalent only when the GMR and 90% CI were within 80–125%, and all other results were considered non-BE. To analyze the reasons for the non-BE of the rapidly eliminated BCS class I drugs DEX, it is important to distinguish between the results of the BE experiment that “demonstrate BE,” “fail to demonstrate BE,” “fail to demonstrate BIE,” and “demonstrate BIE” (Figure 1).

Thus, in this study, we aimed to determine whether the current biowaiver standard is reasonable for DEX. First, a PBPK model of DEX was established and used to evaluate the effects of dissolution, permeability, liquid gastric emptying, and solid gastric emptying time on the C_max_ and area under the curve calculated up to the last simulated time point (AUC_tlast_) of DEX. Second, to assess the risk associated with applying the biowaiver procedure (drugs showed not bioequivalent *in vivo* but have similar dissolution behavior *in vitro*), we simulated the success ratio of virtual BE under the biowaiver boundary conditions. This mechanism-based simulation can reduce the risk that needs to be taken when deciding on the biowaiver of BCS class I drugs.

## Materials and Methods

### Development of the PBPK Model and Selection of Parameters

**FIGURE 2:**
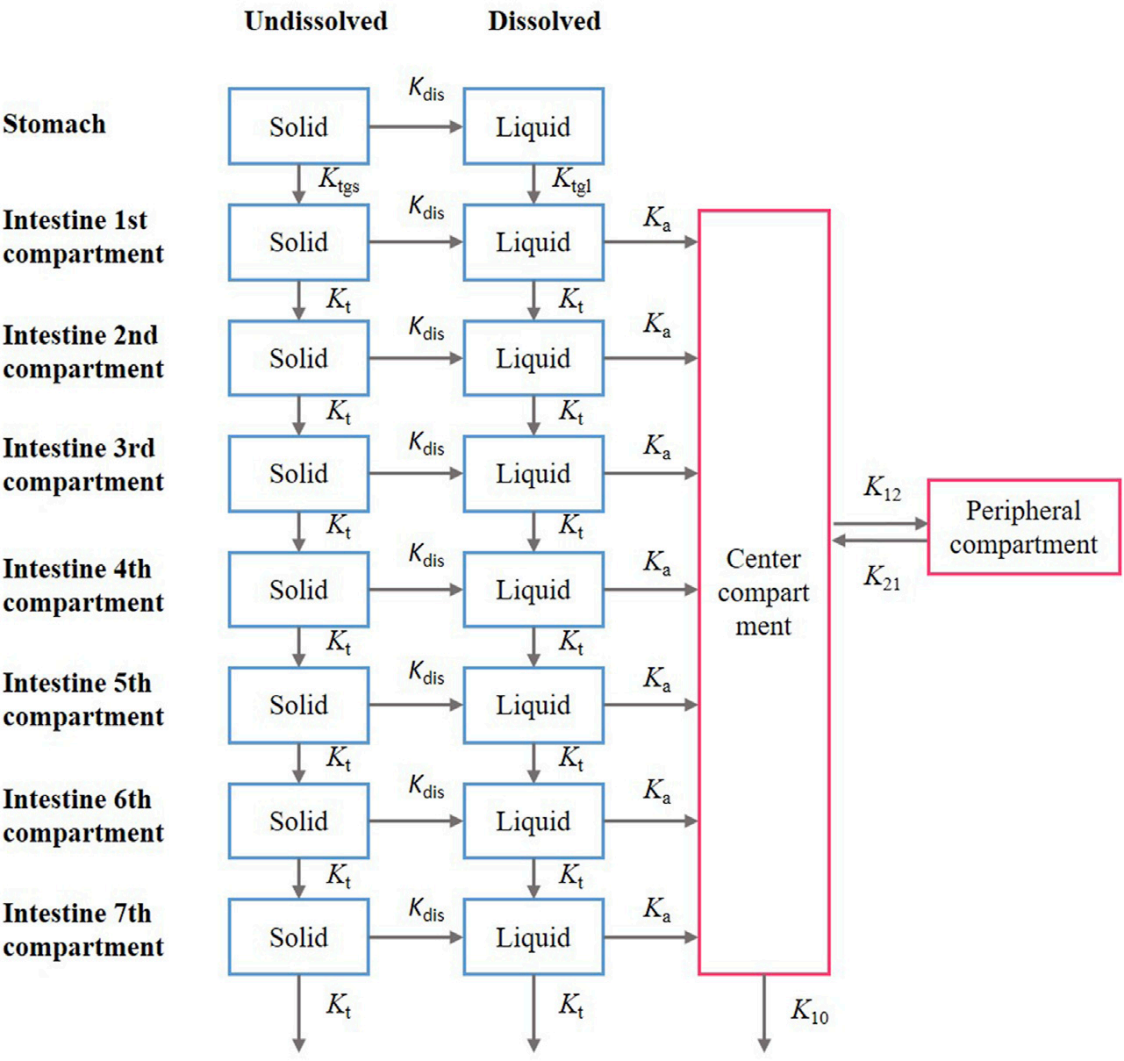
Structure of the compartmental absorption and transit (CAT) model and two- compartment model; the parameter used in the models include: *K*_dis_ is the dissolution rate constant; *K*_a_ is the absorption rate constant; *K*_tgl_ is the liquid gastric emptying rate constant, *K*_*t*gs_ is the solid gastric emptying rate constant; *K*_t_ is the intestinal gastric emptying rate constant; *K*_12_ is the transit rate constant from the central compartment to the peripheral compartment; *K*_21_ is the transit rate constant from the peripheral compartment to the central compartment; *K*_10_ is the first-order elimination rate constant.

In this model, the gastrointestinal tract was considered to comprise one gastric segment and seven intestinal segments, with each segment divided into two compartments, representing undissolved and dissolved drugs. The structure of the PBPK model is presented in (Figure 2). The drug absorption is described according to the compartmental absorption and transit model (Yu et al., 1996; Kortejärvi and Aurtti, 2007), and the distribution and elimination of the drug are described using a two-compartment model. Drug was administered to the stomach compartment, dissolved in the stomach and intestines, but could only be absorbed in the intestinal compartment. The dissolution and absorption processes were assumed to obey first-order kinetics. The differential equations describing this model are presented below.

$$\frac{dA_{\text{s}\left(\text{gas}\right)}}{dt}=-K_{\text{tgs}}\times A_{\text{s}\left(\text{gas}\right)}-K_{\text{dis}}\times A_{\text{s}\left(\text{gas}\right)}$$ (1)

$$\frac{dA_{\text{l}\left(\text{gas}\right)}}{dt}=-K_{\text{tgl}}\times A_{\text{l}\left(\text{gas}\right)}+K_{\text{dis}}\times A_{\text{s}\left(\text{gas}\right)}$$ (2)

$$\frac{dA_{\text{s}\left(\text{n}\right)}}{dt}=K_{\text{t}\left(\text{n}-1\right)}\times A_{\text{s}\left(\text{n}-1\right)}-K_{\text{t}\left(\text{n}\right)}\times A_{\text{s}\left(\text{n}\right)}-K_{\text{dis}}\times A_{\text{s}\left(\text{n}\right)}$$ (3)

$$\frac{dA_{\text{l}\left(\text{n}\right)}}{dt}=K_{\text{t}\left(\text{n}-1\right)}\times A_{\text{l}\left(\text{n}-1\right)}-K_{\text{t}\left(\text{n}\right)}\times A_{\text{l}\left(\text{n}\right)}+K_{\text{dis}}\times A_{\text{s}\left(\text{n}\right)}-K_{\text{a}}\times A_{\text{l}\left(\text{n}\right)}$$ (4)

where, *A*_s(gas)_ is the amount of undissolved drug in the stomach, *A*_l(gas)_ is the amount of dissoved drug in the stomach, *A* is related to the amount of drug in each intestinal segment, the subscripts l and s are related to compartments with dissolved and undissolved drug, respectively, and n is related to gastrointestinal segments. *K*_dis_ is the dissolution rate constant, *K*_a_ is the absorption rate constant, *K*_tgl_ is the liquid gastric emptying rate constant, *K*_tgs_ is the solid gastric emptying rate constant, *K*_t_ is the intestinal transit rate constant, *K*_12_ is the transit rate constant from the central compartment to the peripheral compartment, *K*_21_ is the transit rate constant from peripheral compartment to the central compartment, and *K*_10_ is the first-order elimination rate constant. The parameter values and references are presented in Table 1.

**TABLE 1:** Summary of input parameters used in the dexketoprofen PBPK model.

Parameter (Unit)	Value	References/Comments
Dose (mg)	25	Barbanoj et al. (2001)
Caco-2 cell *P*_app_ (×10^−6^ cm/s)	81.60	Laitinen et al. (2003)
Predicted *P*_eff,man_ (cm/h)	3.37	Predicted by Simcyp^®^ Simulator, version 18
*K*_dis_ (1/h)	8.16 (pH 1.2)	Curve fit
	6.59 (pH 4.5)	Curve fit
	8.29 (pH 6.8)	Curve fit
*K*_tgs_ (1/h)	1.65	Wilding et al. (2003)
*K*_tgl_ (1/h)	5.67	Calculated^a^
*K*_t_ (1/h)	2.10	Yu et al. (1996)
V_c_ (L)	3.55	Fitted by Phoenix WinNonlin (7.0) software
*K*_12_ (1/h)	1.14	Fitted by Phoenix WinNonlin (7.0) software
*K*_21_ (1/h)	1.24	Fitted by Phoenix WinNonlin (7.0) software
*K*_10_ (1/h)	1.68	Fitted by Phoenix WinNonlin (7.0) software
A (mg/L)	11.04	Fitted by Phoenix WinNonlin (7.0) software
B (mg/L)	3.20	Fitted by Phoenix WinNonlin (7.0) software
Alpha (1/h)	3.45	Fitted by Phoenix WinNonlin (7.0) software
Beta (1/h)	0.60	Fitted by Phoenix WinNonlin (7.0) software

a Mean value of 10,000 times gastric emptying model simulation results.

The intestinal transition time has been reported to be approximately 3.3 h (Yu et al., 1996), and the intestinal transit rate is independent of the dosage form. Therefore, we assumed that the drug is transported in the intestine at a uniform rate, and that the solid intestinal transit rate constant is equal to the liquid intestinal transit rate constant (Christensen et al., 1985). The mean liquid and solid gastric emptying rate constants were 5.67 and 1.65 L/h, respectively (Wilding et al., 2003). Based on this first-order process of gastric emptying, the liquid gastric emptying rate constant (*K*_tgl_) was calculated using the following equations (Tsume and Amidon, 2010):

$$T_{1\text{/}2}=GET\times \ln\left(2\right)$$ (5)

$$K_{\text{tgl}}=\frac{\ln\left(2\right)}{T_{1\text{/}2}}$$ (6)

where *T*_1/2_ is the time required to empty half of the stomach contents and *GET* is the gastric emptying time.

Using the absorption module in SimCYP^®^ software (Version 18.0) to predict *P*_eff_ in human and the *K*_a_ of DEX was calculated using the following equation:

$$K_{\text{a}}=\frac{2\times P_{\text{eff}}}{\text{R}}$$ (7)

In the above equation, R is the intestinal radius. The *P*_eff_ of DEX in humans is 3.37 cm/h, which is higher than the *P*_eff_ value of metoprolol (0.54 cm/h) (Kim et al., 2006), indicating that DEX has high permeability. The dissolution data were from Garcia-Arieta et al.’s reports (Garcia-Arieta et al., 2015), and used in our PBPK model. Apparatus 2 (paddle apparatus) was used at 75 rpm, and various dissolution media were used at 900 ml and pH 1.2, 4.5, and 6.8. The dissolution in media with a pH of 1.2, 4.5, and 6.8 was set as the dissolution in the stomach, the first intestinal segment (duodenum), and other intestinal segments in our model (Garcia-Arieta et al., 2015), respectively (Supplementary Figure S1). More than 99% of DEX is bound to plasma proteins and is excreted mainly through urine after extensive metabolism (González-Canudas et al., 2019). Pharmacokinetic parameters were evaluated *via* compartmental modeling using Phoenix WinNonlin (Version 7.0) after intravenous (i.v.) bolus administration in humans (Valles et al., 2006). The *in vivo* verification data are the plasma concentration-time curve data of oral 37 mg of DEX (equivalent to 25 mg of dexketoprofen) (Barbanoj et al., 1998) and the data of oral 18.5 mg of DEX (equivalent to 12.5 mg of dexketoprofen) (Mauleón et al., 1996) under fasting conditions.

The model was built using the Berkeley Madonna software (Version 8.3.18), with the Runge–Kutta 4 integration method. The goodness-of-fit of the developed oral absorption PBPK model was assessed by the percentage prediction error (%PE) of C_max_ and AUC_tlast_ as follows:

$$\%\text{PE}=\frac{\left(\text{Observed Value }-\text{Predicted Value}\right)}{\text{Observed Value}}\times 100$$ (8)

### Parameter Sensitivity Analysis

Parameter sensitivity was analyzed to assess the importance of the selected input parameters in oral absorption. Using the model, we simulated the dissolution rate under pH 1.2, 4.5, and 6.8 conditions, intestinal permeability, liquid gastric emptying time, and solid gastric emptying time. Pharmacokinetic profile simulations were performed by changing the reference parameter value of the drug from 33.3 to 300% of the baseline value. C_max_ and AUC_tlast_ values were obtained from these simulations. Next, the changes in parameter values were normalized to the baseline values of the reference drug, and the changes in C_max_ and AUC_tlast_ were normalized to the values of baseline C_max_ and AUC_tlast_, respectively. If the dissolution rate constant was lower than 3.8 h^−1^ and the permeability lower than 0.54 cm/h, the simulation was abandoned because it would exceed the boundary of the BCS class I. The parameter range is shown in Table 2.

**TABLE 2:** Selected model parameters and the ranges in sensitivity analysis.

	Low	Baseline	High
Dissolution rate in pH 1.2 media (h^−1^)	3.8	8.16	24.48
Dissolution rate in pH 4.5 media (h^−1^)	3.8	6.59	19.77
Dissolution rate in pH 6.8 media (h^−1^)	3.8	8.29	24.87
*P*_eff_ (cm/h)	0.54	3.37	10.11
Liquid gastric emptying rate (h^−1^)	1.89	5.67	17.01
Solid gastric emptying rate (h^−1^)	0.55	1.65	4.95

### Virtual BE Simulation

To assess the BIE risk factors for BCS class I drugs due to high variations in gastric emptying, we established a gastric emptying variation model. Oberle et al. published data (Oberle et al., 1990) on 1) the gastric emptying rate constant and delay time after taking 200 ml and 50 ml of water at different gastric motility periods in the fasting state, and 2) the distribution of each cycle of the migrating myoelectric complex (MMC). The model used in the present study was based on these data and was able to predict the variations in gastric emptying.

In the MMC cycle, the duration of phases I, II, and III and the complete cycle showed logarithmic normal distribution, the mean and standard deviation (SD) values were 46 ± 24 (*n* = 25), 107 ± 68 (*n* = 22), 8.1 ± 4.3 (*n* = 35), and 151 ± 69 (*n* = 20) min, respectively. The mean ± SD of the log(lengths) were 1.6 ± 0.26, 1.9 ± 0.34, 0.81 ± 0.24, and 2.13 ± 0.22 for phases I, II, and III, and the complete MMC cycle, respectively (Oberle et al., 1990). A comparison of the rate and distribution of MMC in the gastric emptying variation model with previously reported values is shown in Supplementary Figures S2, S3, and Supplementary Table S1. The gastric emptying parameters at each stage were recorded with an average distribution of ±2 SD. For the same subject, the duration of the gastric emptying period was identical. We compared the results of the model run 1,000 times with the published literature values containing individual liquid gastric emptying data. We compared the mean liquid gastric emptying half-life values with three previous reports (Adkin et al., 1995; Basit et al., 2001; Hens et al., 2014). The results of this model are not significantly different from the published values (Supplementary Table S2). The gastric emptying variation model describes the variation of gastric emptying time between individuals, and the randomness between two sequences of drug administration.

By changing the dissolution rate of the test formulation, virtual BE experiments were simulated to explore the BIE risk of DEX under the current biowaiver guidance. According to the results of the sensitivity analysis, we determined that the dissolution rate under pH 4.5 and 6.8 hardly affected pharmacokinetic (PK) parameters *in vivo*; thus, we only simulated the virtual BE for changes in the dissolution profile under pH 1.2 condition. We used the goal seek method to obtain two dissolution curves with a similarity factor (f2, calculate by Equation 9) value of 50 (FDA., 2017). The two dissolution curves indicates that the boundary dissolution rate meets the BCS-based biowaiver requirements. The boundary was defined as the dissolution profile of fast- and slow-dissolution formulation. The dissolution rate of the formulation with medium dissolution was equal to that of the original formulation. These borders corresponded to the dissolution safe space of biowaiver guidance. We used these borders to predict the result of BE studies to assess the risk of biowaiver. By introducing the variations in gastric emptying time and randomness to the virtual BE test, the plasma concentration time profiles of 24 and 48 subjects were simulated separately. The virtual BE trials were designed as two-sequence, two-treatment, two-period, crossover studies. Virtual BE studies were simulated 1,000 times between formulations with fast, medium, and slow dissolution with the reference formulations. We calculated the pharmacokinetic parameters (C_max_ and AUC_tlast_) and 90% CI to determine whether BE Statistical analysis of the virtual BE test results was conducted using Rstudio 1.0.143 (Rstudio, Boston, Massachusetts).

$${\text{f}}_{2}=50\times \log\left\{{\left[1+\left(1/n\right)\sum_{t=1}^{n}{\left(R_{t}-T_{t}\right)}^{2}\right]}^{-0.5}\times 100\right\}$$ (9)

## Results

### Model Validation

**FIGURE 3:**
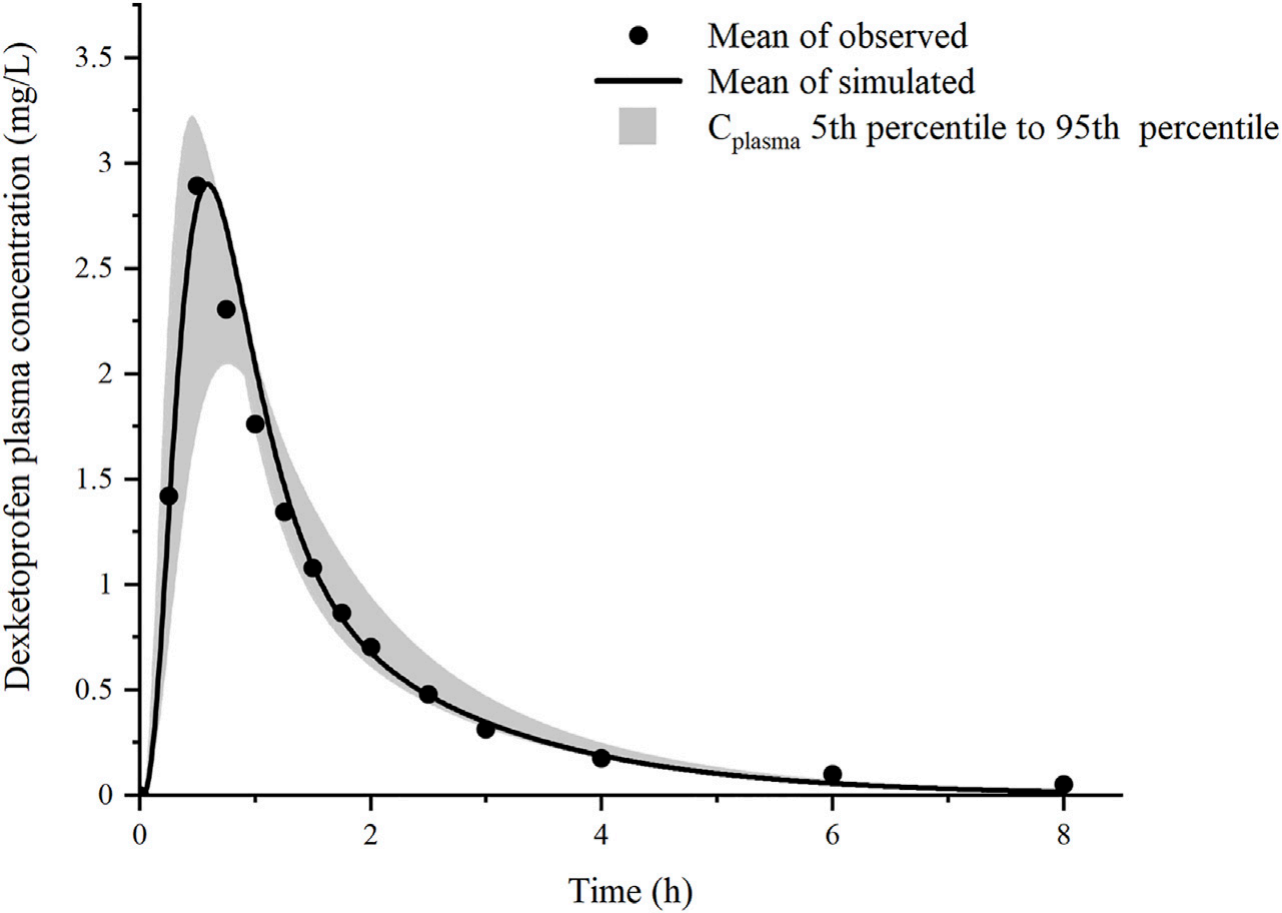
Prediction results of the mean plasma concentration of dexketoprofen after oral administration of 37 mg dexketoprofen trometamol (corresponding to 25 mg dexketoprofen) (black line), dexketoprofen plasma concentration (solid circles, *n* = 18), and the result of 1,000 simulations after the addition of the gastric emptying variation model (gray shaded region).

The predicted plasma drug concentration profiles of DEX well matched the observed values (Figure 3, Supplementary Figure S4 and Supplementary Table S3). The PBPK model was evaluated by performing simulation to assess the C_max_ and AUC_tlast_ of 25 and 12.5 mg DEX tablets compared with the observed data. The predicted PK parameters were consistent with the observed data (Supplementary Table S3).

### Sensitivity Analysis of Parameters That Affect Plasma Drug Levels

**FIGURE 4:**
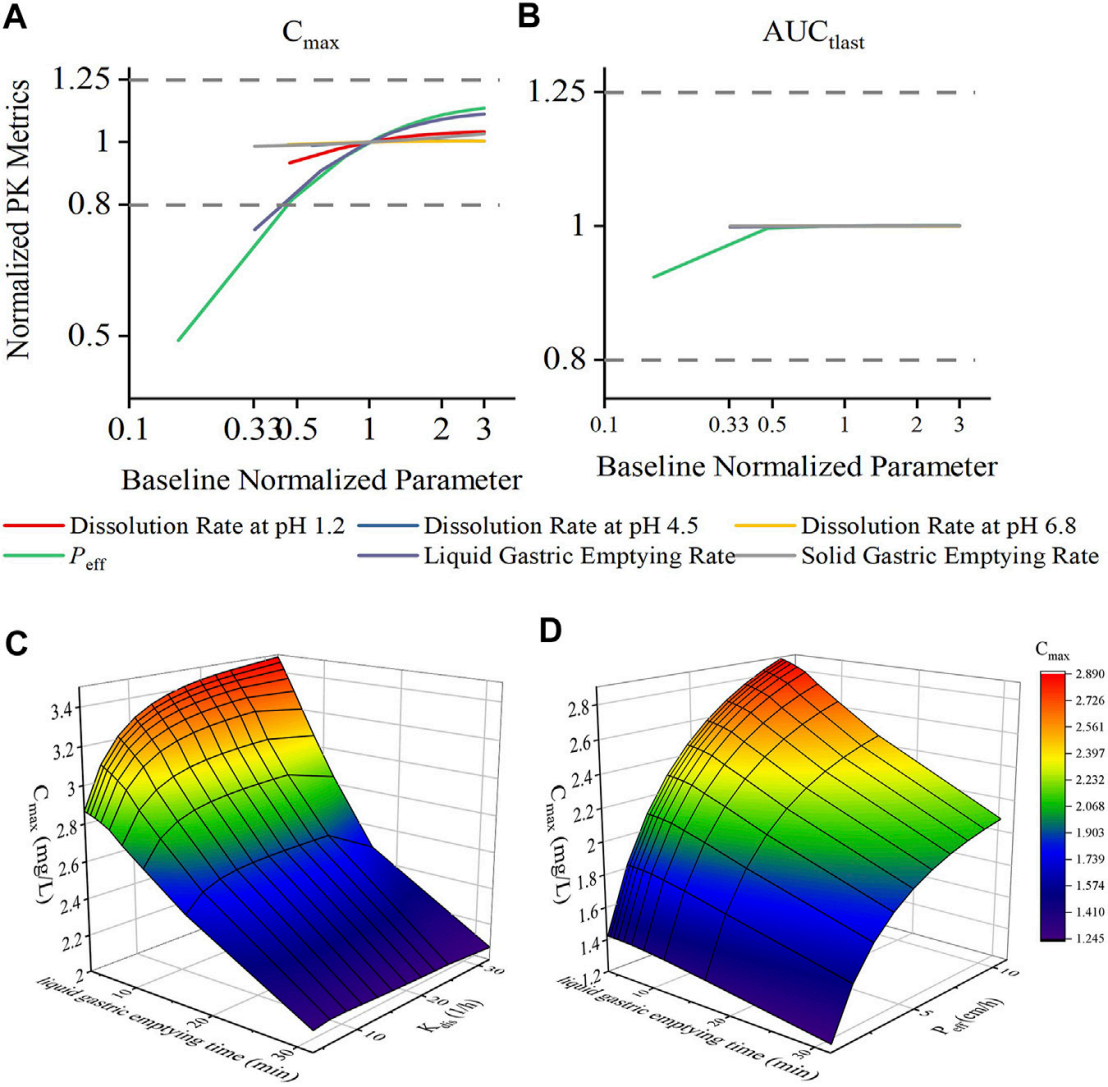
Effect of changes in drug permeability, dissolution, liquid gastric emptying time, and solid gastric emptying time on the C_max_ and AUC_tlast_ changes for dexketoprofen, and the results of plasma profiles of various parameters simulated by the PBPK model. Changes in parameters were normalized to the baseline value of reference value, whereas the C_max_ and AUC_tlast_ changes were normalized to the baseline C_max_ **(A)** and AUC_tlast_ **(B)** values and the BE limits of 80–125% boundary for the C_max_ and AUC_tlast_ (dashed lines), respectively. 3D surface response plot to show the relationship of dexketoprofen liquid gastric emptying time (min) with dissolution **(C)** and absorption **(D)** effects on the C_max_.

Sensitivity analysis was performed to robustly investigate the effects of dissolution, permeability, and gastric emptying time on the pharmacokinetics of DEX. As shown in Figures 4A, B, the ratio of C_max_ was generally more sensitive than that of AUC_tlast_ for these parameters; in fact, AUC_tlast_ was almost unaffected by these factors owing to complete absorption. For *K*_dis_, the dissolution rate at pH 1.2 had a greater impact on C_max_, and the dissolution rate at pH 4.5 and 6.8 had almost no effect on C_max_. For *P*_eff_, C_max_ and AUC_tlast_ were different; changing the values of *P*_eff_ in the range of 0.54–3.37 cm/h led to a two-fold change in the C_max_ ratio. Similar trends were observed for the liquid gastric emptying rate. When the value of *K*_tgl_ was changed from 1.89 to 17.01 h^−1^, C_max_ increase with *K*_tgl_, from 2.10 to 3.33 (mg/L), and the C_max_ ratio dropped below 80%. For the solid gastric emptying rate, when the *K*_tgs_ value was changed in the range of 0.55–4.95 h^−1^, the C_max_ ratio remained in the range of 80–125%. To better understand the effect of gastric emptying time on the plasma concentration of BCS class I drugs, a relationship was established between gastric emptying with dissolution and absorption, and its impact on C_max_ and AUC_tlast_ (Figures 4C, D). The surface response plot suggested that the changes in gastric emptying time lead to substantial changes in the C_max_ of rapidly dissolved drugs, but led to marginal changes in the C_max_ of slowly dissolved drugs. Similar trends were observed in the relationship between gastric emptying and drug absorption times.

### Virtual BE

**FIGURE 5:**
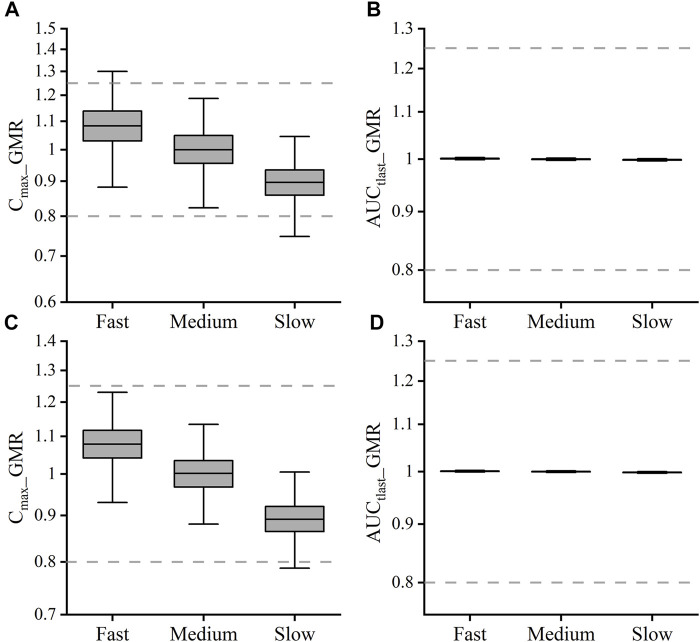
Distribution of the GMR in 1,000 virtual BE simulations, **(A)**: C_max_ (24 subjects in each trial); **(B)**: AUC_tlast_ (24 subjects in each trial), **(C)**: C_max_ (48 subjects in each trial); **(D)**: AUC_tlast_ (48 subjects in each trial).

To evaluate the change in dissolution rate to the BE results, fully replicated, two-sequence, two-treatment, two-period, crossover virtual BE studies were simulated. We set the test formulations as fast-, medium- (which had the same dissolution rate as the reference formulation), and slow-dissolution formulations (Supplementary Figure S5). Virtual BE results demonstrated that DEX with *in vitro* dissolution reaching 85% dissolved within 30 min would lie within the bioequivalence limits for AUC_tlast_ and GMRs were almost distributed around 100% (Figures 5B, D). For C_max_, the probability of success for bioequivalence of fast and slow formulations was significantly lower than that of the medium formulation (Figures 5A and C). 60% of the fast-dissolution formulation BE test, 85.5% of the medium-dissolution formulations BE test and 52.8% of the slow-dissolution formulations BE test result would lie within the BE limits for C_max_ (Demonstrate BE). In addition, 37.0% of the fast-dissolution formulation BE test, 14.4% of the medium-dissolution formulations BE test, and 43.6% of the slow-dissolution formulations BE test was summarized as “Fail to Demonstrate BE”. However, the ratio of result showed “Fail to Demonstration BIE” for fast-, medium-, and slow-dissolution formulations was relatively low, only 3.0, 0.1, and 3.6%, respectively. When the sample size of subjects in BE test was enlarged to 48, the ratio of result showed “non-BE” for fast-, medium-, and slow-dissolution formulations significantly decreased to 11.0, 0.6, and 19.6%, respectively, indicated that 89, 99.4 and 80.4% of the trials have passed the bioequivalence test. Besides, The portion of “Fail to Demonstrate BIE” results for each formulation grow to 9, 0.6 and 9.6% respectively (Table 3, Supplementary Figure S6).

**TABLE 3:** Results for C_max_ in virtual BE trials simulated by the DEX PBPK model.

Subjects number	Demonstrate BE		Non-BE							
			Fail to demonstrate BE		Fail to demonstrate BIE		Demonstrate BIE		Total	
	24 (%)	48 (%)	24 (%)	48 (%)	24 (%)	48 (%)	24 (%)	48 (%)	24 (%)	48 (%)
Fast	60.0	89.0	37.0	10.8	3.0	0.2	0.0	0.0	40.0	11.0
Median	85.5	99.4	14.4	0.6	0.1	0.0	0.0	0.0	14.5	0.6
Slow	52.8	80.4	43.6	18.6	3.6	1.0	0.0	0.0	47.2	19.6

## Discussion

**FIGURE 6:**
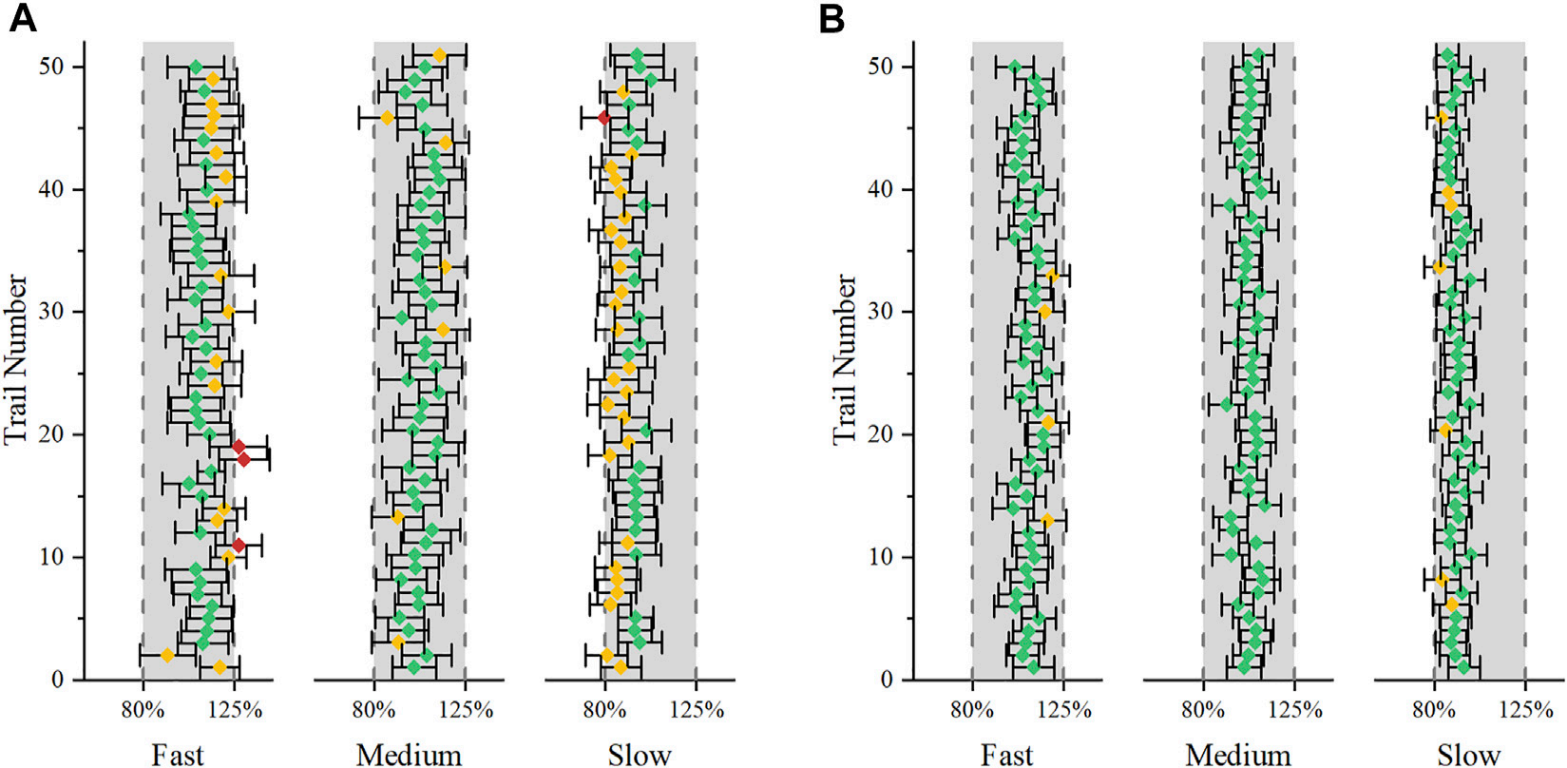
The first 50 virtual simulation in the 1,000 virtual BE trials. Error bars represent the 90% confidence intervals. Green indicates that the results successfully showed BE, yellow indicates that the results failed to show BE, and red indicates that the results failed to show BIE. **(A)**: 24 subjects were included; **(B)**: 48 subjects were included.

In this paper, modeling and simulation methods are used to predict the drug concentration profile *in vivo* when the *in vitro* dissolution of the generic formulation is similar to the reference formulation, and to predict the successful possibility of the *in vivo* BE test by carrying out a large number of virtual BE tests. The present virtual BE studies indicated that although the fast- and slow-dissolution formulations showed a high non-BE probability, the ratio of failed to show BIE result, which can prove the difference between the two formulations, was not high (Table 3) (Granero et al., 2006). When the GMR is between 80–125%, the sampling deviation of the equivalence test can be reduced by increasing the number of samples, but when the GMR exceeds 80–125%, the success rate of bioequivalence cannot be improved by increasing the number of samples. When the point was estimated at 110% and the intra-individual variation was 25%, 48 subjects were required to maintain the power of the BE test at 80%, as calculated using the PowerTOST package of R software. When the number of subjects was increased to 48 subjects, the proportion of non-BE results dropped to an acceptable level. To better understand the results of C_max_, we plotted the results of the first 50 virtual simulations in 1,000 virtual BE trials separately (Figure 6). In the virtual trial simulation of 24 subjects, only four trials failed to show BIE among the non-BE trials. When the subject number was increased to 48, no result failed to show BIE. Increasing the number of sample cases can reduce false negative results, and the results indicating that the risk of therapeutic alternatives of products within the scope of biowaiver guidance and original research products was low. According to the literature published by Alfredo Garcia-Arieta et al. (2015) the number of subjects in the four failed BE trials were 29, 42, 22, and 34, respectively. However the coefficient of variation intrasubiects CV (%) are 29.68, 30.84, 24.61 and 27.75, the sample size was obviously insufficient. This can also explain part of the reason for the failure of the BE test.

**FIGURE 7:**
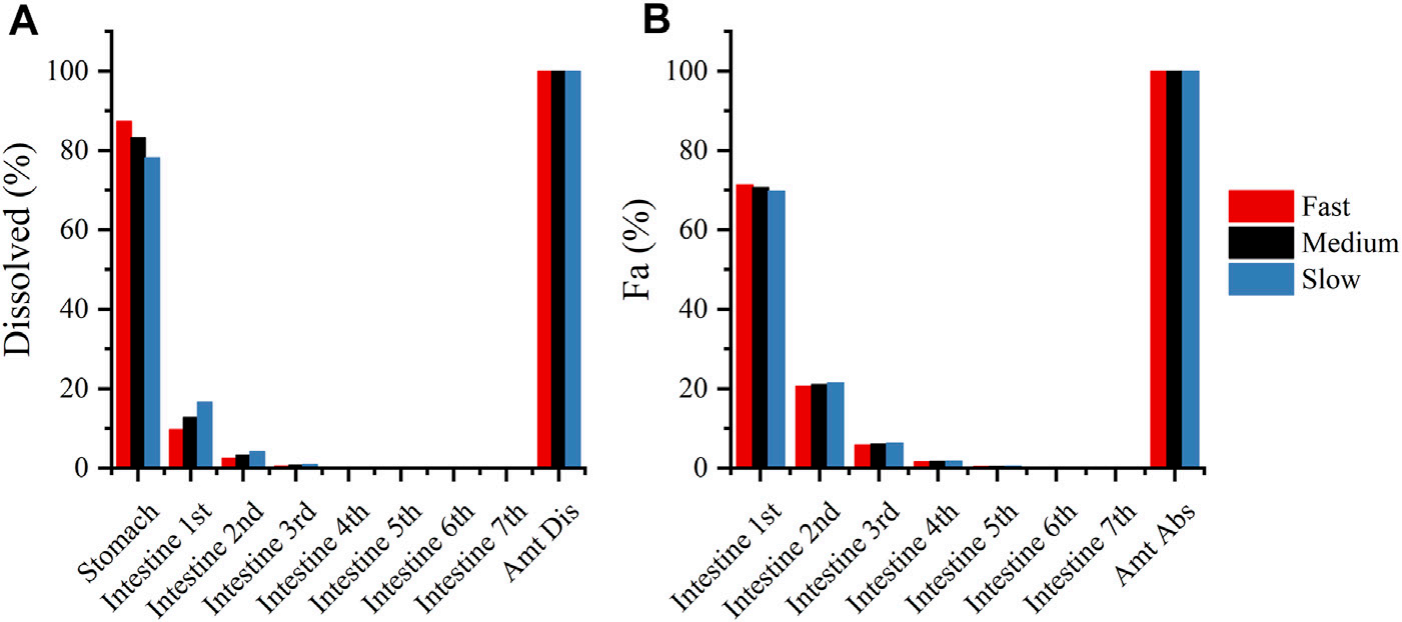
**(A)**: Compartmental dissolution of the three DEX formulations in the PBPK model; **(B)**: compartmental absorption of the three DEX formulations in the PBPK model.

Although the current sensitivity analysis is rough, it is still valuable for analyzing the rate-limiting steps that affect DEX absorption. The physiological and pharmaceutical significance of these parameters should be carefully evaluated in real-world settings. Sensitivity analyses revealed that the dissolution rate in pH 1.2 buffer media may affect the absorption of DEX, compared with the dissolution in pH 4.5 and 6.8 buffer media. (Figure 7) shows the fraction of dissolved and absorbed drugs in each gastrointestinal segment for the fast-, medium-, and slow-dissolution formulations. More than 75% of the drugs were dissolved in the stomach, and more than 70% of the drugs were absorbed in the first intestinal segment. This may explain why the dissolution rate in pH 1.2 buffer media was important. Therefore, in the study of virtual BE, we only changed the dissolution rate in pH 1.2 buffer media.

Permeability and liquid gastric emptying time are also sensitive factors for DEX absorption. Gastric emptying time is a highly variable physiological parameter (Oberle et al., 1990). Simulation of the interaction between gastric emptying time with dissolution and absorption times showed that drugs with rapid dissolution and absorption would lead to a greater change in C_max_. Therefore, we added the simulation of gastric emptying variation in the PBPK model of DEX. In the guidelines issued by the FDA (FDA, 2017), the biowaiver of BCS class Iproducts was more relaxed than that of BCS class III drugs, and it is reported that when new excipients or atypically large amounts of commonly used excipients are included in an IR solid dosage form, additional information documenting the absence of an impact on BA of the drug may be requested by FDA (FDA, 2017). In fact, only a few excipients have been reported for their effect on gastric emptying time. Most relevant research focused on the effect of excipients on intestinal transit time and intestinal permeability (Flanagan, 2019). Thus, there is a lack of research on the effect of common excipients on gastric emptying time. However, the biowaiver guidelines of each regulatory agency have requirements for excipients. The recently released ICH (International Conference on Harmonization of Technical Requirements for Registration of Pharmaceuticals for Human Use) M9 guideline clearly states that the possible effects of excipients on *in vivo* absorption parameters, such as solubility, gastrointestinal motility, transit time, intestinal permeability, and transporter mechanisms, should be considered (ICH, 2019). In particular, for drugs that are sensitive to gastric emptying, such as DEX, it is necessary to consider whether excipients affect gastric emptying time, especially if new excipients or atypically large amounts of common excipients are used. Therefore, in future studies, we will investigate the effect of some commonly used excipients on gastric emptying time. If any excipients that affect gastric emptying are used in the BCS Class I drugs with rapid elimination, the approval of the biowaiver needs to be carefully considered.

The excipients of the DEX reference products include microcrystalline cellulose, maize starch, glycerol distearate, and sodium starch glycolate (Garcia-Arieta et al., 2015). There is a lack of research on the effects of these excipients on intestinal permeability. Lovering et al. reported that the permeability coefficient of diazepam was unaffected by lactose, microcrystalline cellulose, and starch (Lovering et al., 1976). Dahlgren et al. pointed out that the effects of excipients shown in models without normal intestinal transport may overestimate the potential of excipients to affect permeability (Dahlgren et al., 2018). The pharmacokinetics of excipients and their local luminal concentration in the intestine have not been investigated thus far. Thus, considering that the permeability coefficient is not a highly variable parameter, permeability was not simulated and discussed fully. This is a limitation of the current study.

The drug disposition was simulated by two-compartment model. If there is enough human data *in vivo*, PBPK can be considered as a disposition model. This can introduce *in vivo* physiological parameters, such as metabolic enzyme expression, body weight, and body surface area et al. as analysis objects. However, the parameters in the disposition model and absorption model may affect the drug concentration level. Complicating the model may increase the difficulty of identifying sensitive factors and establishing model parameter requirements. The complexity and simplification of the model are determined by the ultimate purpose of the research. For this research the chemical structure of the drug determines its pharmacokinetics and the generic product has the same qualitative and quantitative composition in active substances as the reference product. Therefore, using the two-compartment model not only simplifies the PBPK model but also satisfies the research purpose.

## Conclusion

In the present study, we observed that although the existing literature shows that the C_max_ of DEX is prone to lead to non-BE results, DEX is not a drug with a high risk of biowaiver. According to our research, formulations with different dissolution rates within the boundaries of the biowaiver were bioequivalent to the original drugs. As a sensitive factor, the influence of excipients on liquid gastric emptying should be further studied to determine whether the current guidelines for biowaiver are reasonable for excipients. The established PBPK model is a convenient tool to evaluate the dissolution, permeability, and gastric emptying time, which affect the pharmacokinetic parameters of the drug. It can be applied to the risk assessment of biowaivers to reduce the possibility of waiver failure.

## Data Availability

The original contributions presented in the study are included in the article/Supplementary Material, further inquiries can be directed to the corresponding author.

## Abbreviations

AUC_tlast_, area under the curve calculated up to the last simulated time point; BA, bioavailability; BCS, Biopharmaceutics Classification System; BE, bioequivalence; BIE, bioInequivalence; CI, confidence interval; C_max_, peak plasma drug concentration; DEX, dexketoprofen trometamol; f2, similarity factor; GMR, geometric mean ratio; MMC, migrating myoelectric complex; Non-BE, Non-bioequivalence; *P*_app_, apparent permeability coefficient; *P*_eff_, the effective intestinal permeability; PBPK, physiologically based pharmacokinetic; PK, pharmacokinetic; T_max_, the time to maximal plasma concentration.
