# Supplementary material for: A Physiologically Based Pharmacokinetic Model for Studying the Biowaiver Risk of Biopharmaceutics Classification System Class I Drugs With Rapid Elimination: Dexketoprofen Trometamol Case Study
Source: Front Pharmacol. 2022 Feb 10;13:808456. doi: 10.3389/fphar.2022.808456 (PMC8904038; doi:10.3389/fphar.2022.808456)
Supplement: Supplementary file 1 [file DataSheet1.PDF]

Electronic Supplementary Material

A Physiologically Based Pharmacokinetic Model for Studying the Biowaiver Risk of

Biopharmaceutics Classification System Class I Drugs with Rapid Elimination:

Dexketoprofen Trometamol Case Study

Xian Zhang, Xuxiao Ye, Kuan Hu, Wenping Li, Wenqian Li, Qingqing Xiao, Lin Chen, Jin Yang

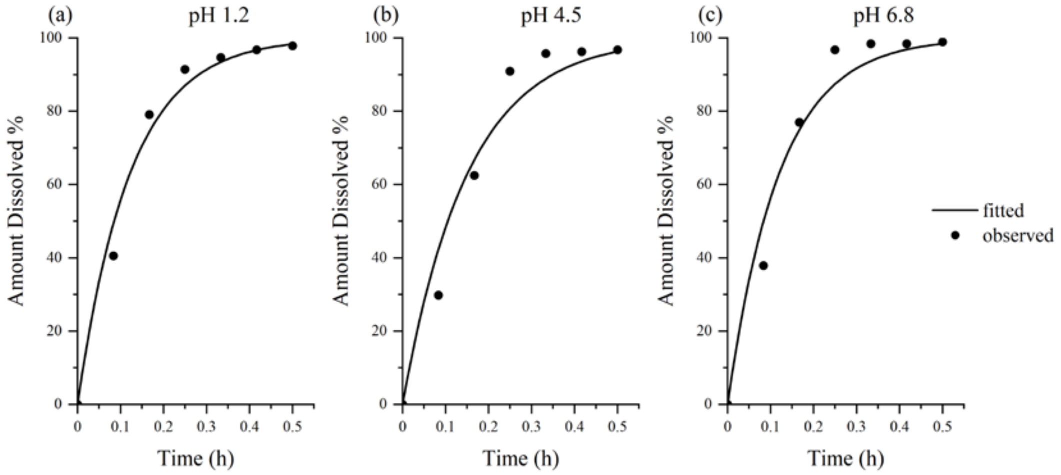

Figure s1. Observed (points) and curve data (lines) of dexketoprofen Trometamol at (a) pH 1.2 conditions (b) pH 4.5 conditions (c) pH 6.8 conditions in vitro

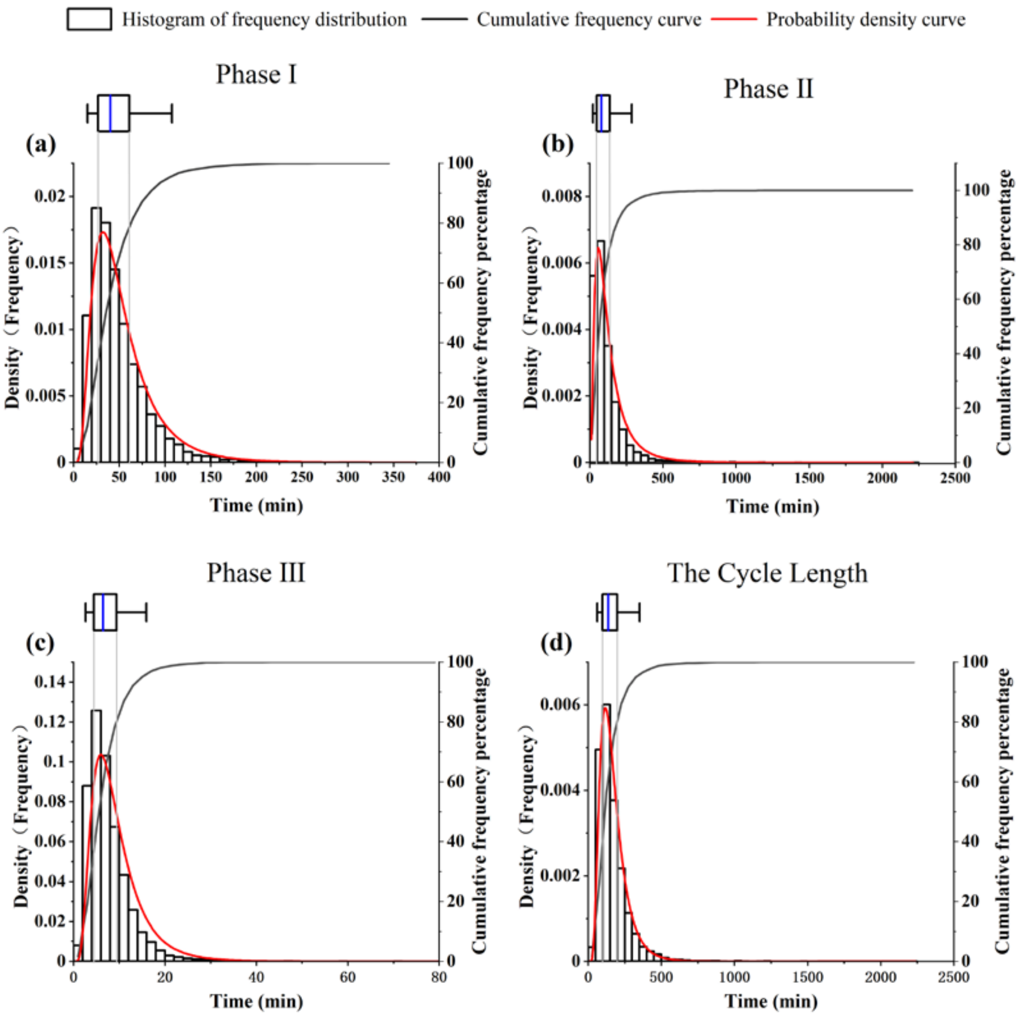

Figure s2. Distribution of phase I (a), phase II (b), phase III (c), and the complete cycle length (d) time in the migrating myoelectric complex (MMC) cycle. Statistics of the results of the model run 10,000 times and the histogram is the frequency histogram of the results. Above the histogram is a box plot of the results, showing the 5% quantile, 25% quantile, 75% quantile, and 95% quantile for 10,000 results. The graph plots are cumulative distribution curve of the simulation results (black) and the published (Oberle et al., 1990) probability density curve (red) consistent with the simulation results

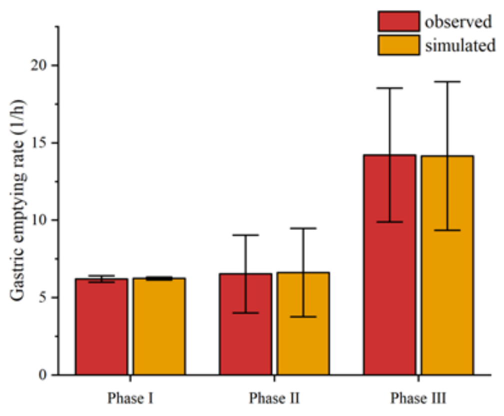

Fig. s3. Gastric emptying rate published (observed) and simulated by the model (simulated)

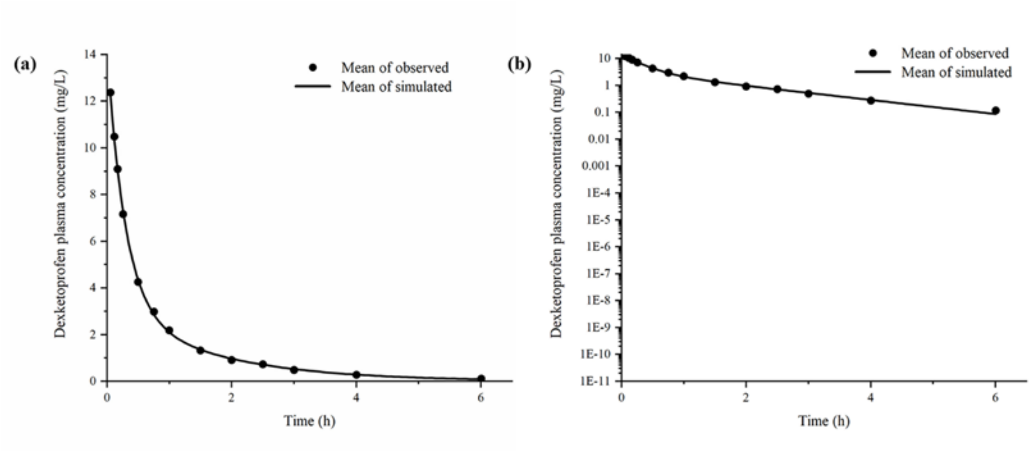

Fig. s4. Prediction results of the mean plasma concentration of dexketoprofen after i.v. bolus administration of 37 mg dexketoprofen trometamol (corresponding to 25 mg dexketoprofen) (black line), dexketoprofen plasma concentration (solid circles, n = 12)

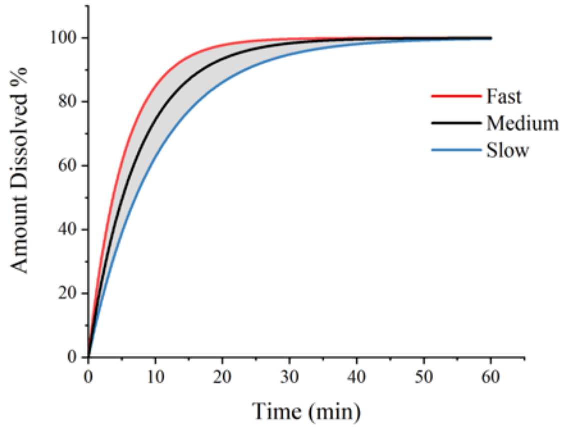

Fig. 5. Dissolution profiles of DEX formulations with fast, medium, and slow dissolution rate in pH 1.2 media

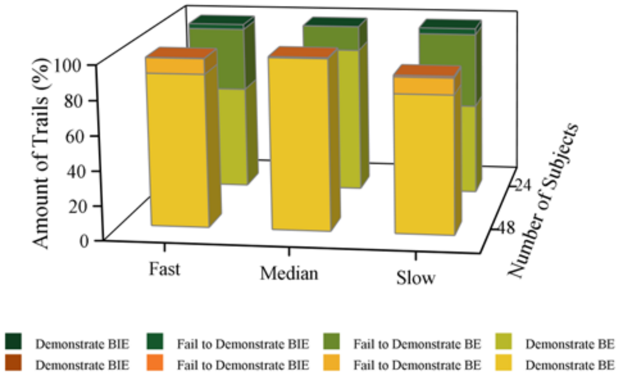

Fig. s6. Results of a virtual BE trial study using PBPK when 24 subjects and 48 subjects were enrolled

Table sⅠ. Summary of MMC stages in the literature and the 10,000 simulations in the dexketoprofen PBPK model

Phase	Data from literature[a]	Simulated data
Phase I	46 ± 24 (n = 25)	47.8 ± 31.5 (n = 24000)
Phase II	107 ± 68 (n = 22)	107.9 ± 98.4 (n = 24000)
Phase III	8.1 ± 4.3 (n = 35)	7.5 ± 4.4 (n = 24000)
MMC cycle[b]	151 ± 69 (n = 20)	163.2 ± 103.7 (n = 24000)

[a]Reference 19

[b]MMC, migrating myoelectric complex

Table sⅡ. Summary of average liquid gastric emptying half-life values simulated by the gastric emptying variation model and literature data

Data source	Liquid gastric emptying half-life (h)	References
Simulation	0.122	
Literature	0.087	(Hens et al., 2014)
Literature	0.229	(Basit et al., 2001)
Literature	0.183	(Adkin et al., 1995)

Table sⅢ. Comparison of DEX pharmacokinetic parameters from published and simulated data

Pharmacokinetic parameter	25 mg			12.5 mg		
	Simulated	Observed	PE%	Simulated	Observed	PE%
C_{max} (mg/L)	2.90	3.10	6.5%	1.45	1.38	5.1%
AUC_{tlast} (mg/L·h)	4.17	4.03	3.6%	2.09	1.66	25.7%
